# Proteomics-based identification of different training adaptations of aged skeletal muscle following long-term high-intensity interval and moderate-intensity continuous training in aged rats

**DOI:** 10.18632/aging.102044

**Published:** 2019-06-26

**Authors:** Fang-Hui Li, Lei Sun, Da-Shuai Wu, Hao-En Gao, Zhu Min

**Affiliations:** 1 School of Sport Sciences, Nanjing Normal University, Nanjing, China

**Keywords:** aging, skeletal muscle, proteome, high-intensity interval training, adiponectin

## Abstract

Aging-associated loss of skeletal muscle mass and force increases the risk of falls, impairs mobility, and leads to a reduced quality of life. High-intensity interval training (HIIT) is superior to moderate-intensity continuous training (MICT) for improving morphological and metabolic adaptations of skeletal muscle in older adults, but the underlying mechanism is unknown. Aged female rats underwent HIIT and MICT for 8 months, and their differential impacts on skeletal muscle proteome were investigated. HIIT resulted in a larger improvement in grip strength and fiber cross-sectional area, with similar increases in inclined plane performance and time to exhaustion. Proteomic analysis showed that common training adaptations of both protocols included changes to muscle contraction, focal adhesion signaling, mitochondrial function, apoptosis and regeneration, and anti-oxidation, whereas protein processing in the endoplasmic reticulum and adipocytokine signaling were specifically altered in the MICT and HIIT groups, respectively. Immunoblotting showed that upregulation of the adiponectin/AMPK signaling pathway may be associated with improvements in autophagy, oxidative stress, mitochondrial function, and apoptosis in aged skeletal muscle following HIIT. Thus, understanding the molecular differences in training adaptations from these two exercise modalities may aid in combatting sarcopenia.

## INTRODUCTION

Sarcopenia, the natural course of age-associated loss in muscle mass and function, can be mitigated, at least partially by physical activity [1]. The health benefits of exercise in combating sarcopenia are indisputable, and understanding the transducers of such benefits is of much interest [2]. Recently, high-intensity interval training (HIIT) has received attention owing to its physiological and psychological benefits in the elderly [3], especially in combating sarcopenia [4–6]. HIIT is considered more enjoyable than traditional, moderate-intensity continuous training (MICT), and leads to improved performance, muscle protein synthesis, and muscle contractile function [4, 5, 7]. Moreover, 12 weeks of HIIT reverses the age-related differences in the skeletal muscle proteomes of older humans, particularly with increased synthesis of mitochondrial proteins involved in mitochondrial function and hypertrophy [4]. Previously, we observed that HIIT initiated late in life in an aged rat model offered more benefits than MICT in combating age-induced chronic low-grade inflammatory status and oxidative stress in skeletal muscles [7]. However, a comprehensive approach to long-term HIIT programming and the mechanism of training skeletal muscle adaptations remain to be determined.

Comprehensive proteomic analysis provides insights into the structural composition and functional alterations in skeletal muscle in response to exercise [8]. Until now, only four studies have examined the global effects of exercise on aged skeletal muscle plasticity using high-throughput proteomic techniques. Brocca et al. and Cobley et al. examined the effects of endurance training on the myofibrillar proteome in older humans using label-free liquid chromatography-tandem mass spectrometry (LC-MS/MS) [9, 10]. Robinson et al. investigated the impact of 12 weeks of HIIT, resistance training, and combined exercise training on skeletal muscle in older humans using LC-MS/MS and showed that the protein composition of older skeletal muscles differed in key energy metabolism enzymes following different exercise programs, demonstrating the scope of label-free mass spectrometry-based approaches in proteomic muscle profiling, especially for metabolic enzymes [4]. Fewer data exist on the long-term effects of HIIT, particularly changes in the protein profiles of skeletal muscles in an aged rat model.

In this study, a label-free proteomics approach using LC-MS/MS was applied to investigate protein changes in skeletal muscles following 8 months of HIIT or volume-matched MICT compared with those in untrained aged rats. The aim was to obtain comprehensive insights into the differential beneficial effects of long-term HIIT and MICT on aged skeletal muscle. Furthermore, we aimed to identify the changes in muscle composition depending on training status that might be relevant to prevent and/or treat sarcopenia.

## RESULTS

### Physical performance and muscle fiber morphology

**Figure 1:**
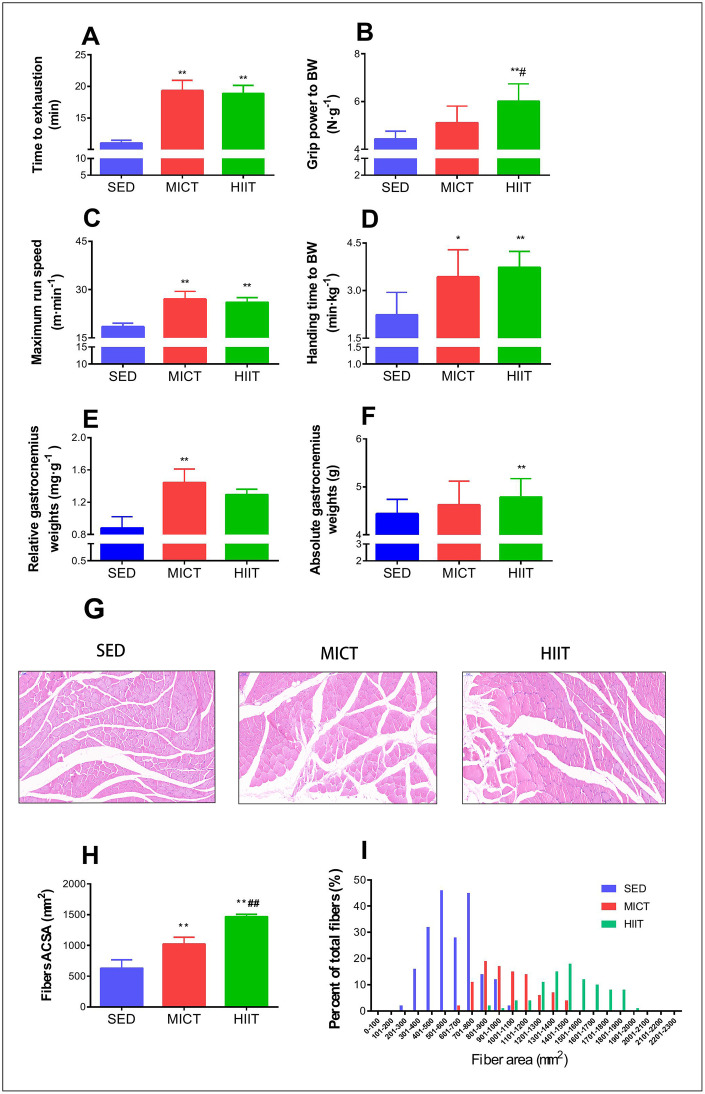
**Effect of MICT and HIIT protocols on physical performance and fiber cross sectional area.** Changes in time to exhaustion (min) (**A**), grip strength (N·g^–1^) (**B**), maximum run speed (m·min^-1^) (**C**), inclined plane performance (**D**), relative gastrocnemius weight (**E**), absolute gastrocnemius weight (**F**), muscle fiber morphology (**G**), mean cross-sectional area (**H**), and frequency distribution of mean gastrocnemius fiber cross sectional area (**I**). SED, sedentary control; MICT, moderate-intensity continuous training; HIIT, high-intensity interval training; BW, body weight. Data were analyzed by one-way ANOVA followed by Tukey’s post-hoc test and are presented as mean ± SD. ^*^ p < 0.05 *vs*. SED; ^**^ p < 0.01 *vs*. SED; ^#^ p < 0.05 *vs*. MICT.

The inclined plane performance, grip strength, maximum running speed, and run time to exhaustion in the rats from the three groups after 8 months of exercise training are shown in Figure 1. Both HIIT and MICT rats showed improvements in running time to exhaustion (HIIT: p < 0.001, MICT: p < 0.001, Figure 1A), maximum running speed (HIIT: p < 0.001, MICT: p < 0.001, Figure 1C), and inclined plane performance (HIIT: p < 0.001, MICT: p < 0.05, Figure 1D) compared to those in SED rats. HIIT animals showed better grip power performance than both SED (p < 0.001, Figure 1B) and MICT rats (p < 0.05; Figure 1B), but no significant changes in grip power were apparent between MICT and SED rats (p > 0.05, Figure 1B).

As shown in Figure 1E–1F, MICT rats showed a significant increase in relative gastrocnemius weight compared to that in the SED group rats (p < 0.01). HIIT animals showed a significant increase in absolute gastrocnemius weight (p < 0.01), but the relative gastrocnemius weight was not significantly greater than that in SED rats. Both HIIT and MICT resulted in a significant increase in the mean CSA compared with that in the SED group (p < 0.01; Figure 1H), whereas HIIT animals exhibited a significantly higher mean CSA than MICT rats (p < 0.01; Figure 1H).

### Alterations in red gastrocnemius muscle proteome

**Figure 2:**
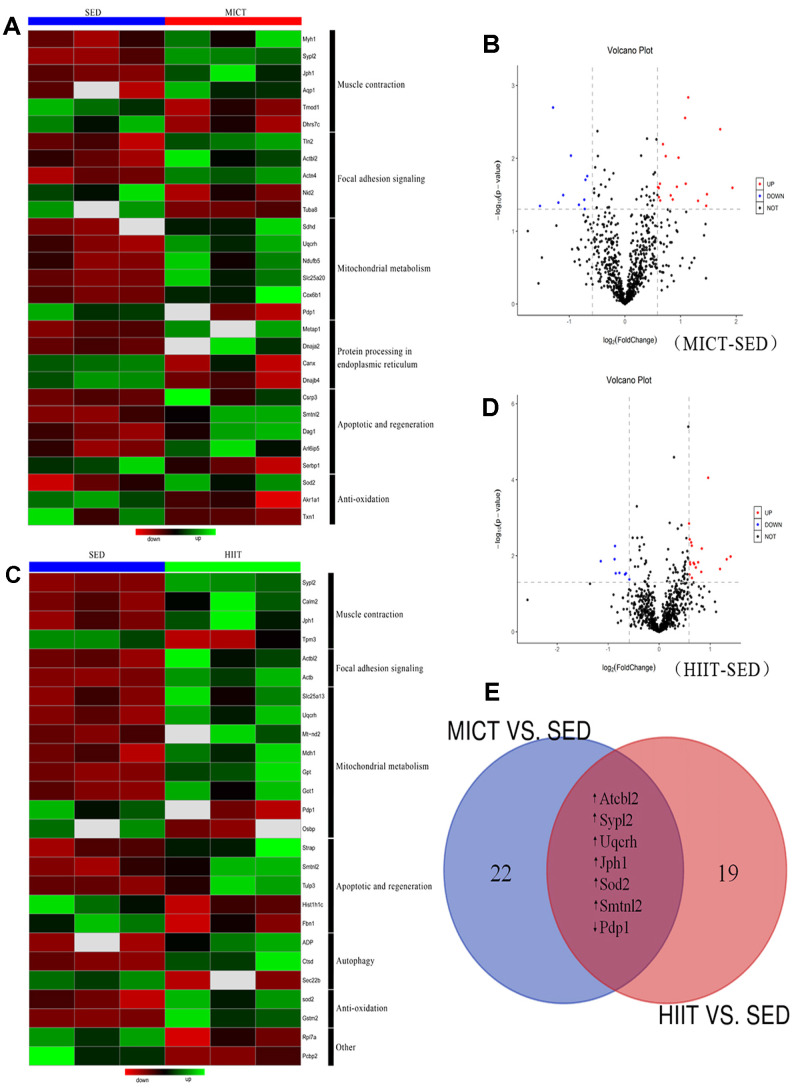
**Relative protein abundances in the red gastrocnemius muscle of rats in the MICT, HIIT, and SED groups.** (**A**) Heat map and (**B**) volcano plot of significantly up- and downregulated proteins in the red gastrocnemius muscle from MICT and SED rats (p < 0.05, fold change > ± 1.5). (**C**) Heat map and (**D**) volcano plot of significantly up- and downregulated proteins in the red gastrocnemius muscle from HIIT and SED rats. (**E**) Overlap of proteins up- and downregulated following different modes of exercise training compared with their levels in the SED group.

A label-free proteomics approach using LC-MS/MS was applied to investigate protein changes in the following two comparisons: (i) MICT *vs*. SED and (ii) HIIT *vs*. SED (Figure 2). With a 1% false-discovery rate (FDR), 823 and 814 proteins were identified in the first and second comparisons, respectively. Among these, 29 and 26 differentially expressed proteins (DEPs) were identified in the MICT and HIIT groups compared with the levels in the SED group, respectively (Figure 2A–2D; Supplementary Tables 3, 4). Gene ontology (GO) classification revealed that most DEPs in both datasets were involved in similar functions (Figure 2A and 2C), indicating the presence of commonly altered functional pathways within the red gastrocnemius in the MICT and HIIT rats. Furthermore, 7 proteins were observed in both DEP datasets (Figure 2E): six of these were upregulated, whereas one was downregulated compared to the levels in the SED group.

### Functional classification of DEPs

Among the 29 DEPs in the MICT group (Supplementary Table 3), six were related to muscle contraction, two of which were downregulated [tropomodulin 1 (Tmod1) and dehydrogenase 7C (Dhrs7c)], and four were upregulated [myosin heavy chain 1 (Myh1), synaptophysin-like 2 (Sypl2), junctophilin 1 (Jph1), and aquaporin 1 (Aqp1)]. Five DEPs were related to cell integrity, with two downregulated [nidogen 2 (Nid2) and tubulin alpha 8 (Tuba8)] and three upregulated [talin 2 (Tln2), actin beta-like 2 (Actbl2), and actinin alpha 4 (Actn4)]. Six DEPs were related to mitochondrial metabolism, including one that was downregulated [pyruvate dehydrogenase phosphatase catalytic subunit 1 (Pdp1)] and five that were upregulated [succinate dehydrogenase complex subunit D (Sdhd), ubiquinol-cytochrome c reductase hinge protein (Uqcrh), NADH:ubiquinone oxidoreductase subunit B5 (Ndufb5), solute carrier family 25 member 20 (Slc25a20), and cytochrome c oxidase subunit 6B1 (Cox6b1)]. Four DEPs were related to protein processing in the endoplasmic reticulum (ER), including two downregulated [calnexin (Canx) and DnaJ heat shock protein family (Hsp40) member B4 (Dnajb4)] and two upregulated DEPs [methionyl aminopeptidase 1 (Metap1) and DnaJ heat shock protein family (Hsp40) member A2 (Dnaja2)]. In addition, five DEPs were involved in apoptosis and regeneration, of which one was downregulated [serpine1 mRNA binding protein 1 (Serbp1)] and four were upregulated [cysteine and glycine rich protein 3 (Csrp3), smoothelin-like 2 (Smtnl2), dystroglycan 1 (Dag1), and ADP-ribosylation like GTPase 6 interacting protein 5 (Arl6ip5)]. Finally, three DEPs were related to anti-oxidation, including two downregulated [aldo-keto reductase family 1 member A1 (Akr1a1) and thioredoxin 1 (Txn1)] and one upregulated DEP [superoxide dismutase 2 (SOD2)].

Among the 26 DEPs in the HIIT group (Supplementary Table 4), four were related to muscle contraction with one downregulated [tropomyosin 3 (Tpm3)] and three upregulated [calmodulin 2 (Calm2), Sypl2, and Jph1]. Additionally, two upregulated DEPs were related to cell integrity [Actbl2 and actin beta (Actb)]. Eight DEPs were involved in mitochondrial metabolism, including two downregulated [oxysterol binding protein (Osbp) and Pdp1] and six upregulated [Uqcrh, mitochondrially encoded NADH dehydrogenase 2 (Mt-nd2), solute carrier family 25 member 13 (Slc25a13), malate dehydrogenase 1 (Mdh1), glutamic-oxaloacetic transaminase 1 (Got1), and pyruvate dehydrogenase phosphatase catalytic subunit 1 (Gpt)]. Five DEPs were related to apoptosis and regeneration, among which two were downregulated [fibrillin 1 (Fbn1) and histone cluster 1 H1 family member c (Hist1h1c)], and three were upregulated [serine/threonine kinase receptor associated protein (Strap), Smtnl2, and tubby-like protein 3 (Tulp3)]. Three DEPs were related to autophagy, including one downregulated [SEC22 homolog B, vesicle trafficking protein (Sec22b)] and two upregulated DEPs [cathepsin D (Ctsd) and adiponectin (ADP)]. Finally, two upregulated DEPs were related to anti-oxidation [Sod2 and glutathione S-transferase mu 2 (Gstm2)].

### Activation of signaling pathways enriched in DEP datasets

DEPs were further investigated by Kyoto Encyclopedia of Genes and Genomes (KEGG) pathway analysis (Supplementary Table 5). DEPs in the MICT group mapped to two significantly enriched signaling pathways: four upregulated proteins were involved in forkhead box O (FOXO) signaling (p = 0.014), whereas six upregulated proteins were involved in protein processing in the ER (p = 0.037).

DEPs in the HIIT group mapped to two significantly enriched signaling pathways. Three upregulated proteins were involved in the adipocytokine signaling pathway (p = 0.036), whereas three upregulated proteins were involved in FOXO signaling (p = 0.050).

### Verification of protein and mRNA levels

**Figure 3:**
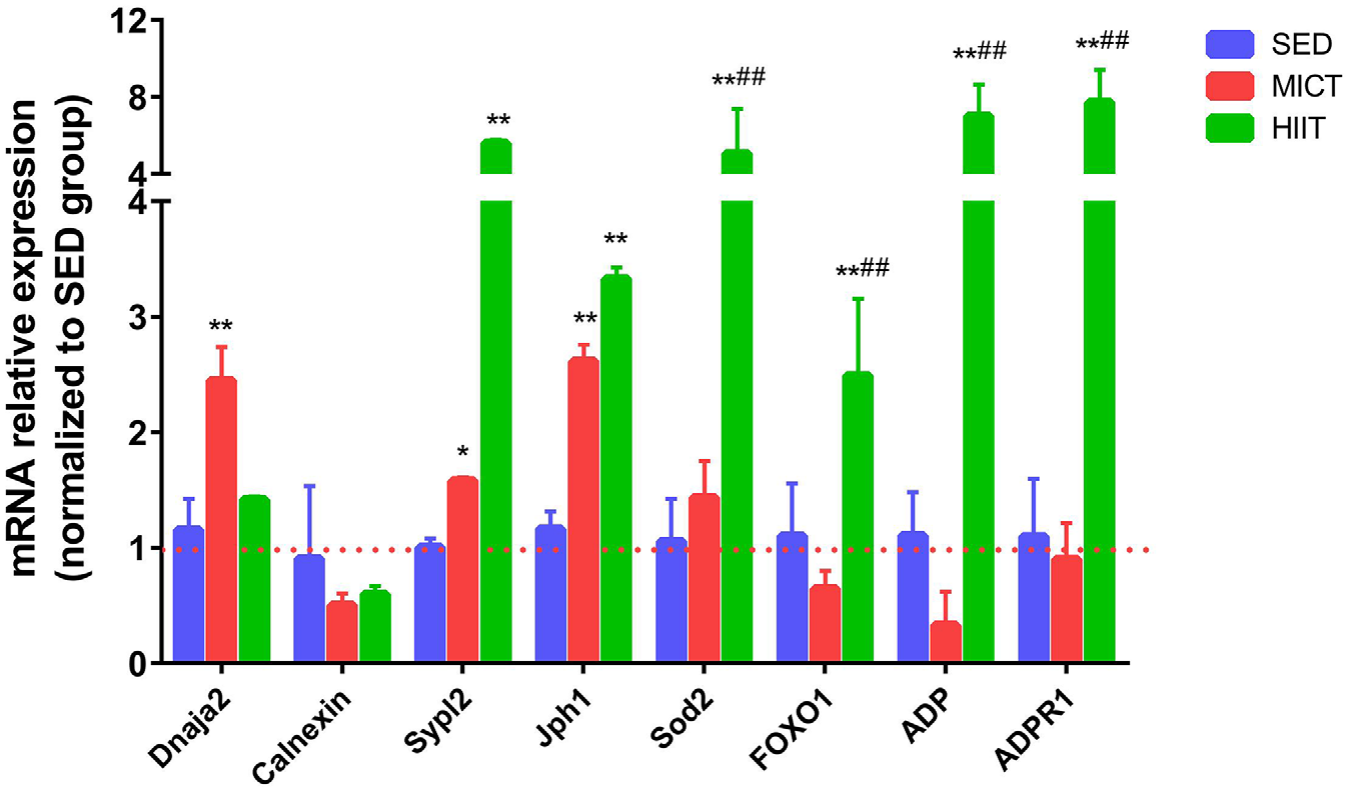
**Evaluation of mRNA levels following HIIT and MICT.** SED, sedentary; MICT, moderate-intensity continuous training; HIIT, high-intensity interval training; DnaJ heat shock protein family (Hsp40) member A2, Dnaja2; Superoxide dismutase 2, Sod2; Junctophilin 1, Jph1; Synaptophysin-like 2, Sypl2; Forkhead box O 1, FOXO1. Data were analyzed by one-way ANOVA followed by Tukey’s post-hoc test and are reported as the mean ± SD. ^*^ p < 0.05 *vs*. SED; ^**^ p < 0.01 *vs*. SED; ^#^ p < 0.05 *vs*. MICT.

According to KEGG analysis, the FOXO signaling pathway was similarly upregulated in both exercise groups compared to that in the SED group, whereas protein processing in the ER and adipocytokine signaling were specifically altered in the MICT and HIIT groups, respectively. The expression of genes involved in processing in the ER, adipocytokine, and FOXO signaling was further investigated by RT-PCR. Figure 3 shows the relative mRNA expression levels of *Dnaja2*, *calnexin*, *Sypl2*, *Jph1*, *Sod2*, *ADP, ADP receptor 1 (ADPR1)*, and *FOXO1* normalized to the reference gene *Gapdh* in both the MICT and HIIT groups *vs*. SED. *Dnaja2* was upregulated by MICT but not significantly altered in the HIIT group. *Sypl2* and *Jph1* were significantly upregulated by both MICT and HIIT compared to the SED group. Levels of *Sod2*, *ADP, ADPR1* and *FOXO1* were significantly upregulated by HIIT compared with those in the MICT group.

**Figure 4:**
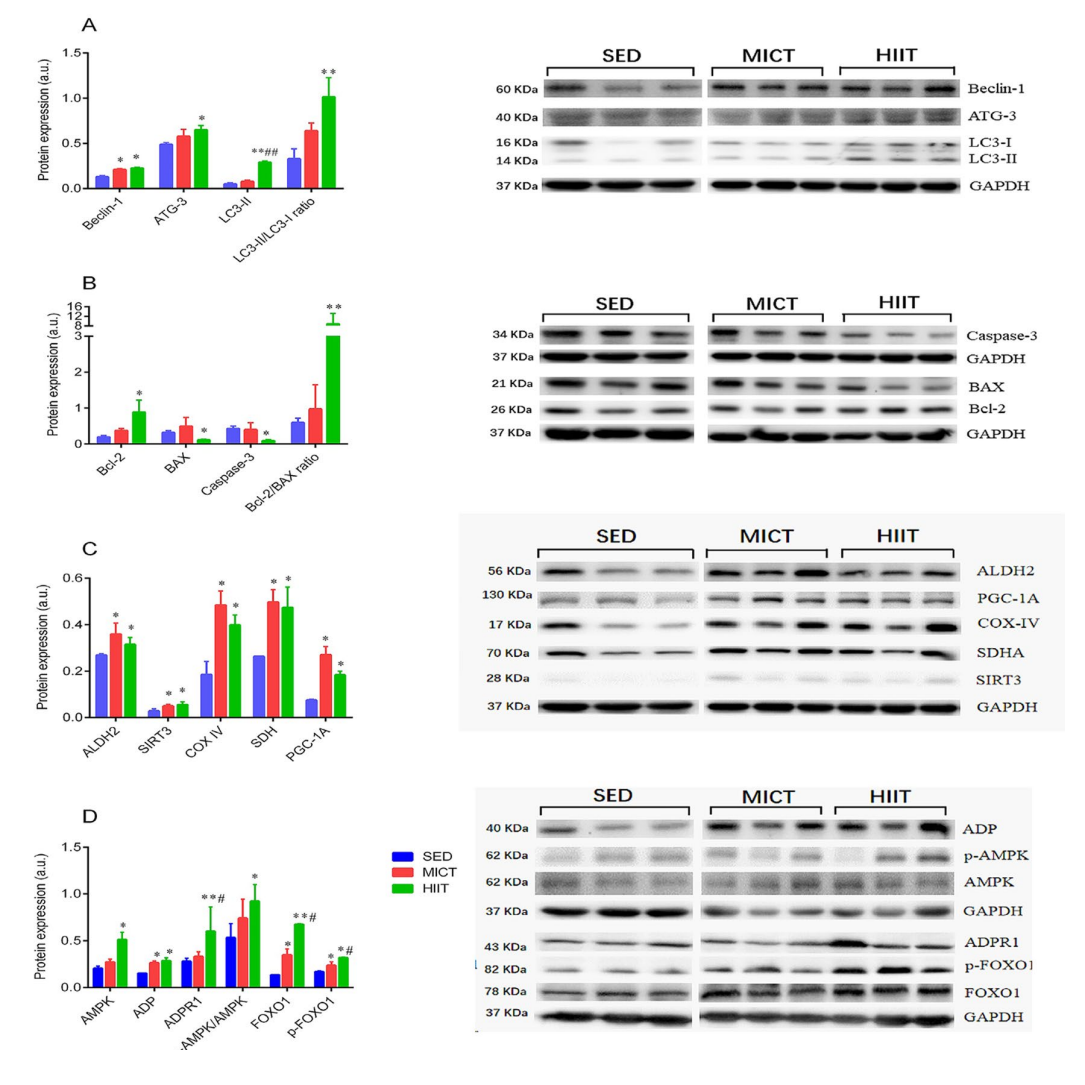
Expression of autophagy (**A**), apoptosis (**B**), and mitochondrial function markers (**C**), and adipocytokine signaling-related proteins (**D**). SED, sedentary; MICT, moderate-intensity continuous training; HIIT, high-intensity interval training; succinate dehydrogenase, SDH; sirtuin 3, SIRT3; aldehyde dehydrogenase 2, ALDH2; peroxisome proliferator-activated receptor γ coactivator-1Α, PGC-1a; adiponectin, ADP; autophagy-related gene-3, Atg-3; microtubule-associated protein 1 light chain 3 II, LC3-II; B-cell lymphoma 2, Bcl-2; Bcl-2-associated X protein, Bax; AMP-activated protein kinase, AMPK; adiponectin receptor 1, ADPR1; Forkhead box O1, FOXO1. Data were analyzed by one-way ANOVA followed by Tukey’s post-hoc and are presented as mean ± SD. ^*^ p < 0.05 *vs*. SED; ^**^ p < 0.01 *vs*. SED; ^#^ p < 0.05 *vs*. MICT; ^##^ p < 0.01 *vs*. MICT.

Next, the expression of proteins involved in the FOXO1 pathway, adipocytokine pathway, autophagy, mitochondrial function, and apoptosis was further verified by western blotting. As shown in Figure 4, Beclin-1, complex-IV (COX-IV), succinate dehydrogenase (SDH), sirtuin 3 (SIRT3), aldehyde dehydrogenase 2 (ALDH2), peroxisome proliferator-activated receptor γ coactivator-1Α (PGC-1a), FOXO1, p-FOXO1, and ADP were significantly upregulated in both the MICT and HIIT groups, whereas autophagy-related gene (Atg)-3, microtubule-associated protein 1 light chain 3-II (LC3-II), the LC3-II/LC3-I ratio, B-cell lymphoma 2 (Bcl-2), the Bcl-2/Bcl-2-associated X protein (Bax) ratio, AMP-activated protein kinase (AMPK), p-AMPK, and ADPR1 were only significantly upregulated in the HIIT group. Levels of caspase-3 and Bax were downregulated in HIIT compared with those in the SED group. Additionally, levels of FOXO1, p-FOXO1, LC3-II, and ADPR1 were significantly upregulated by HIIT compared with those in the MICT group.

## DISCUSSION

We identified altered patterns of protein abundance following HIIT and MICT protocols in aged rats, extending the findings from previous exercise training studies and providing a different global picture of skeletal muscle adaptations that occur after 8 months of HIIT and MICT. Common training adaptations of both protocols included changes to muscle contraction, focal adhesion signaling, mitochondrial function, apoptosis and regeneration, and anti-oxidation. Specific training adaptations included an increase in processing in the ER, contributing to the maintenance of protein homeostasis following MICT, whereas alterations in ADP signaling may be mediated the regulation of autophagy, and may therefore contribute to improvements in grip strength following HIIT. These findings open new avenues for future research, including elucidation of the mechanisms of training adaptations caused by the two exercise protocols and their involvement in maintaining muscle structure and function, resulting in beneficial effects on aged skeletal muscle.

### Focal adhesion signaling

Focal adhesion signaling is important in maintaining muscle morphogenesis and cellular adaptive responses; this is disrupted to such a degree in aged rat skeletal muscles that normal cellular adaptive responses are not sufficient to compensate for the accumulating damage [11]. In the current study, we identified alterations in proteins related to adhesion signaling such as Tln2, Nid2, Tuba8, and Actn4, in MICT group rats. Tln2 serves as a mechanical link between the cytoplasmic integrin tail and the actin cytoskeleton. Nid2, an extracellular integrin-β ligand controls integrin activation by modifying interactions between integrin-β and talin. Tuba8, the most divergent member of the mammalian alpha-tubulin family is involved in the cytoskeleton. Actn4 is associated with alpha-tubulin, which is involved in transcription and splicing. Our data show the specific upregulation of beta-actin protein expression following the HIIT protocol.

Interestingly, upregulation of Actbl2, a newly discovered seventh isoform of actin, was observed following the two exercise protocols. Actbl2 binds gelsolin and is involved in enhancing androgen receptor activity [12], which, along with Actbl2 protein concentration, declines with age and correlates with changes in fiber CSA [13] and skeletal muscle insulin-like growth factor I (IGF-1) expression [14]. We speculate that both exercise protocols enhance Actbl2-gelsolin interactions, potentially contributing to increasing muscle fiber CSA and exerting anabolic effects meditated by IGF-1 upregulation in aged skeletal muscle as per our previous findings [7]. Hence, increases in these focal adhesion signaling proteins may increase cytoskeletal stability, and the morphogenetic or adaptive responses induced by MICT and HIIT in aging skeletal muscle.

### Antioxidant defense systems

Aging-associated oxidative stress occurs when the endogenous antioxidative system is overwhelmed by overproduction of reactive oxygen species (ROS), leading to the oxidation of unsaturated lipids and mitochondrial DNA deletion mutations, which are linked to apoptosis activation, contributing to muscle fiber loss [15]. Our previous findings demonstrated that both exercise modalities similarly decreased 8-hydroxy-2'-deoxyguanosine (8-OHdG), a biomarker of oxidative DNA damage, and increased the activity of SOD2 in aged skeletal muscle [7], which is primarily present in the mitochondria. Moreover, in the present study, the *Sod2* mRNA and SOD2 protein levels were upregulated, following both exercise programs, but the *Sod2* mRNA level did not reach statistical significance in the MICT group. SIRT3, one of the seven sirtuin gene families, deacetylates two critical lysine residues on SOD2 and promotes its protein expression *via* AMPK-dependent increases in PGC-1α [16, 17]. Moreover, PGC-1α upregulation is known to increase FOXO1 target genes, such as SOD2 [18]. Notably, our results revealed the upregulated expression of p-AMPK, SIRT3, and p-FOXO1 levels, consistent with FOXO signaling activation by KEGG analysis, suggesting that SIRT3 expression may be required for exercise-induced SOD2 activation through PGC-1α/FOXO1-dependent control of SOD2 transcription in aged skeletal muscle.

Additionally, oxidation of unsaturated lipids generates reactive aldehydes, which form covalent adducts with lipids such as 4-hydroxynonenal (4-HNE) that are present at high concentrations in skeletal muscle [19]. 4-HNE can be metabolized and detoxified by ALDH2, a mitochondrial enzyme that metabolizes toxic aldehydes in a variety of tissues [20]. Consistently, boosting ALDH2 protein expression *via* transgenic treatment enhances the ability of skeletal muscle to better handle ROS *via* SOD2 activation [21]. In our previous study, both exercise programs significantly inhibited 4-HNE [7]; in the present study, similar elevations in ALDH2 protein content in the red gastrocnemius of aged rats were observed following both exercise programs. Thus, ALDH2 upregulation can also contribute to common antioxidant defense systems following both exercise protocols.

Our data showed that MICT specifically downregulated AKR1A1 and Trx1 in the red gastrocnemius of aged rats*.* AKR1A1, an aldehyde reductase, is critical in the ALDH2-dependent degradation of 4-HNE in the liver and other tissues [22], and Trx1 is a mitochondrial antioxidant protein with a redox-active dithiol. Specifically, upregulation of GSTM2, which protects against lipid peroxidation through GSH conjugation, was observed only in the HIIT group. Notably, our previous study demonstrated that HIIT is more effective than MICT at reducing 8-OHdG and MDA levels in the skeletal muscle [7], which may be related to the specific antioxidant enzyme GSTM2.

### Muscle contraction and calcium signaling

Alterations of calcium ion homeostasis may be involved in the muscle contractile dysfunction that occurs with aging [23]. In the present study, Tpm3 and Calm2 upregulation was observed in HIIT, whereas AQP1, Myh1, Tmod1, and Dhrs7C were upregulated in MICT, and Sypl2 and Jph1 were upregulated in both protocols. These proteins are associated with calcium signaling and muscle contraction. Calm2 and Tpm3 meditate calmodulin-dependent protein kinase and are associated with altered Ca^2+^ sensitivity [24], supporting the hypothesis that one of the beneficial aspects of HIIT in maintaining old muscle fibers is [Ca^2+^]i homeostasis. MICT resulted in downregulation of Dhrs7C, a newly identified NAD^+^/NADH-dependent dehydrogenase expressed in skeletal muscle that functions in repressing Ca^2+^ release from ryanodine receptors [25]. AQP1 belongs to the aquaporin water channel protein family, which facilitates water movement across biological membranes in response to osmotic gradients. The functional importance of AQP1 at the t-tubule may involve maintenance of actin cytoskeleton polarity, which is activated by large ionic fluxes occurring as part of the sodium and calcium exchange during excitation–contraction coupling in skeletal muscle [26].

Additionally, Sypl2 and Jph1 contents were upregulated, consistent with the upregulation of *Sypl2* and *Jph1* mRNA as measured by RT-PCR in aged skeletal muscle following both exercise protocols. Increases in Sypl2 and Jph1 support the possibility that these proteins increase calcium signaling and contractile function; the results of proteomic analysis agree with these data showing similar increases in endurance performance (e.g. the inclined plane performance, maximum running speed, and run time to exhaustion) following both exercise protocols. Taken together, these data suggest that both protocols induced similar changes in calcium signaling and muscle contractile protein expression, which counteracted the age-related changes in excitation–contraction coupling of the aged skeletal muscle.

### Apoptosis and regeneration

Calcium overload may act synergistically with oxidative stress to open the mitochondrial permeability transition pores, releasing cytochrome c into the cytosol and inducing consequent apoptosis and impaired muscle regeneration [27]. In our study, GO analysis identified five proteins related to apoptosis and regeneration in both DEP datasets following MICT. Csrp3 and Dag1, related to skeletal muscle regeneration were upregulated, whereas Arl6ip5 and Serbp1, related to skeletal muscle apoptosis, were upregulated and downregulated, respectively. Csrp3 acts as a mechanical stretch sensor by increasing its mRNA and protein levels in response to contractile activity in skeletal muscle [28], which results in enhanced autophagy and susceptibility to apoptosis [29]. Arl6ip5, an anchor protein that retains interacting proteins in the ER, is suggested to translocate from the ER to the plasma membrane and decrease radical-scavenging activity by inhibiting glutathione synthesis, leading to the activation of cell death signaling [30]. Dystroglycan is an extracellular peripheral membrane glycoprotein that helps maintain normal muscle function; evidence indicates that an increase in muscle-specific Dag1 ameliorates loss of the dystrophin-glycoprotein complex [31], and our data suggest that MICT counteracts age-related impaired muscle regeneration and muscular dystrophy by upregulating Dag1. Serbp1, a plasminogen activator inhibitor 1 (PAI-1) mRNA binding protein that inhibits the stability of *PAI-1* mRNA, causes increases in age-associated muscle atrophy, fibrosis, and impairs muscle regeneration, by binding to its cyclic nucleotide-responsive sequence.

We observed upregulation of Strap by HIIT, along with downregulation of Fbn1, both of which are associated with the transforming growth factor-β (TGF-β) signaling pathway. Strap negatively regulates TGF-β signaling by specifically interacting with the TGF-β receptor and is involved in coordinating the translation of collagen α1 (I) and α2 (I) mRNAs to ensure production of correctly modified and folded type I collagen [32]. Fbn1 is an essential extracellular matrix protein, a major constituent of microfibrils, and an extracellular regulator of TGF-β1 [31]. Meanwhile, our results also showed HIIT-induced upregulation of Tulp3 expression and downregulation of Hist1h1c, both of which are involved in regeneration and apoptosis. Tulp3 is a critical repressor of Hedgehog signaling, which promotes successful regeneration after injury via an increased number of proliferating myogenic cells and newly formed myofibers, as well as enhanced vascularization and decreased fibrosis after injury in the muscles of 24-month-old mice [33]. Moreover, Hist1h1c is a cytochrome c-releasing factor that acts in a Bak-dependent manner, mediated by the mitochondrial apoptotic pathway [34]. These findings are in accordance with the our current finding that HIIT inhibits caspase-3 and Bax expression, which acts as a gateway for a variety of proapoptotic signals, accompanied by increased Bcl-2 expression and the Bcl-2/Bax ratio, resulting in the resistance of aged rat skeletal muscles to age-induced apoptosis following both exercise programs. Additionally, both exercise protocols resulted in similar elevations in Smtnl2 protein expression, which promotes the transition from myoblasts to myotubes in differentiating C2C12 cells by binding with mitogen-activated protein kinases [35]. Taken together, these results suggest that apoptotic and regeneration functions are closely related to the beneficial effects of exercise training on aged skeletal muscle.

### Protein processing in the ER

Cellular senescence is associated with elevated ER stress, dysregulated unfolded protein (UPR) signaling, and altered protein homeostasis, whereas physical training may be a potential strategy for reestablishing ER homoeostasis in the elderly [36]. ER stress involves chaperones that belong to several classical chaperone families, such as heat shock protein (Hsp) 40 and Hsp70. It also involves folding enzymes such as calnexin that are unique. Interestingly, our results showed high levels of DNAJA2 in the MICT group, whereas calnexin and DNAJB4 levels tended to be lower in the MICT group compared with those in SED rats. DNAJB4 and DNAJA2 belong to the DNAJ (HSP40) family of HSPs; they assist Hsp70 in protein folding, transport through membranes, degradation, and escape from aggregation, and are important for maintaining cell viability [37]. Calnexin, a calcium-dependent membrane protein, cooperates with a number of enzymes in the process of assisting glycoprotein folding in the ER and participates in ER protein quality control [38]. Specifically, our data show that *Dnaja2* mRNA expression was significantly upregulated and that of calnexin tended to decrease in the MICT group. Additionally, MetAP1, which cleaves the initiator methionine from about 70% of all newly synthesized proteins in almost all living cells and participates in the process of proteolysis [39], was upregulated in the MICT group. Further, many ER chaperones are calcium-dependent for optimal activity, and oxidative stress triggers an ER-dependent atrophic program when mitochondrial dynamics decrease with aging in sedentary rodents; therefore, reduced ER calcium and elevated in mitochondria-derived ROS will reduce chaperone function and ER folding capacity, thereby causing ER stress and activation of UPR signaling during aging. The trend toward higher activation of the calcium signaling system and mitochondrial SOD2 in MICT at least confirms that MICT can restore the age-related increase in UPR related to the improvements in calcium signaling and blunt oxidative stress induced by aging in rat skeletal muscles. Therefore, these results show that MICT ameliorates age-induced ER stress may contribute to exercise-induced protection against the dysfunctional protein homeostasis in the aged rat model.

### Mitochondrial metabolic enzymes

Mitochondria are crucial for energy production, and several metabolic pathways are integrated within this organelle. Previous studies have shown reduced levels of several key enzymes involved in the TCA cycle, β-oxidation, and oxidative phosphorylation (OXPHOS) in all fiber types during aging, contributing to age-related declines in muscle mass and exercise performance [4]. Unsurprisingly, the present observations that both HIIT and MICT induced increases in OXPHOS proteins indicate that exercise training upregulates mitochondrial respiratory capacity in aged rat skeletal muscle. Specific upregulated proteins involved in mitochondrial oxidative capacity in the MICT group include SDHD, Ndufb5, SLC25A20, and Cox6b1. Ndufb5, a subunit of NADH: ubiquinone oxidoreductase is involved in the assembly and activity of complex I within the OXPHOS. SDHD comprises the D subunit of SDH, a unique membrane-bound enzyme that has dual functions in both the TCA cycle for succinate oxidation and the respiratory chain for electron transfer to the terminal acceptor, ubiquinone. This is consistent with the higher SDH protein expression measured by western blot in the MICT group in Figure 4C. Consistently, a previous study of caloric restriction in aged mice showed that upregulation of Cox6b1, a non-transmembrane subunit of cytochrome oxidase, may help increase mitochondrial respiration and ATP production in liver tissues [40]. Concomitant increases in muscle oxidative capacity and the β-oxidation pathway in elderly subjects after endurance training have been shown in earlier studies [41]. In corroboration of these findings, our data demonstrated that 8 months of MICT may serve as a critical intervention to upregulated SLC25A20, a key molecule that transfers fatty acids across the mitochondrial membrane in β-oxidation, and may contributes to improve the mitochondrial β-oxidation pathway in aged skeletal muscle.

Previous work in older men has revealed that HIIT reverses many age-related differences in the proteome, particularly of mitochondrial proteins in concert with increased mitochondrial protein synthesis [4]. Our current data provide evidence of significant *Nd2* and MDH1 upregulation after HIIT compared to their levels in the SED group. *Nd2 is a* subunit of NADH dehydrogenase, the first enzyme complex of the mitochondrial respiratory chain (complex I) that oxidizes NADH to liberate electrons, supporting the translocation of protons across the inner membrane to create a proton gradient. Consistently, we observed significant increases in the expression of GPT and GOT proteins following HIIT; both enzymes can catalyze the reversible transfer of an amino group from glutamate and oxaloacetate to form α-ketoglutarate, and are involved in replenishing TCA cycle intermediates, suggesting that HIIT may influence the TCA cycle through elevation of α-ketoglutarate in the aged rat skeletal muscle. Interestingly, MDH1 is a subunit of the TCA cycle enzyme malate dehydrogenase, which catalyzes the conversion of oxaloacetate and malate by utilizing the nicotinamide adenine dinucleotide (NAD^+^/NADH) coenzyme system. A decrease in MDH1 and subsequent reduction in the NAD^+^/NADH ratio inhibits SIRT3 deacetylase. We speculated that the HIIT-induced increase in MDH1 protein expression and subsequent increase in the NAD^+^/NADH ratio may exert protective effects against the modulation of age-associated mitochondrial function through the SIRT3/PGC-1a pathway (Figure 4C). which orchestrates metabolic alterations and activates the expression of OXPHOS proteins, e.g. SDH, COX-IV, and ND-2 [16].

**Figure 5:**
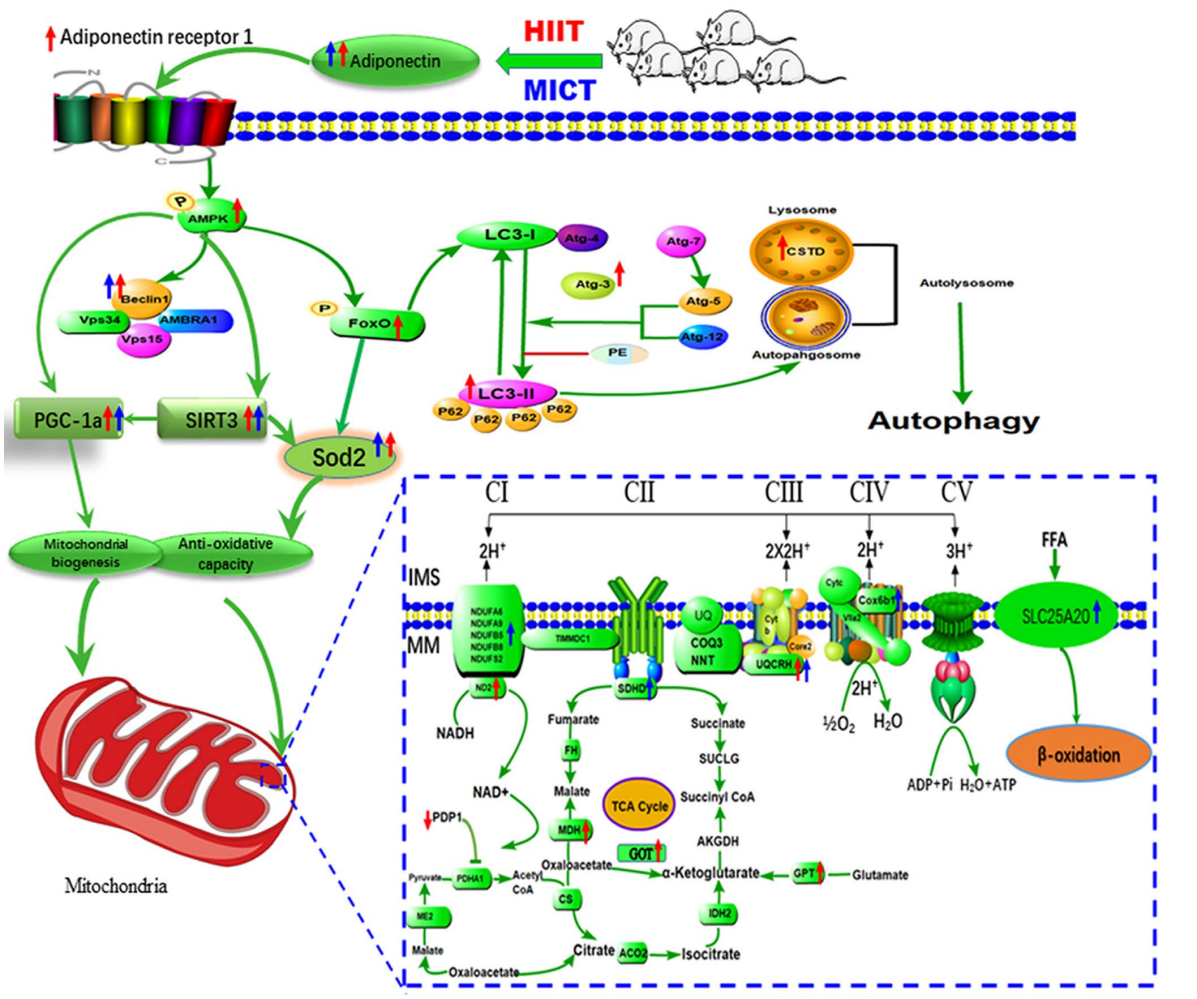
**Proposed model of the mechanism by which HIIT improved skeletal muscle function in aged rats.** HIIT protocols preserve skeletal muscle function by activating lysosomal degradation and improving mitochondrial OXPHOS via the ADP/ADPR1 axis, mediated by the AMPK pathway.

PDP1 and OSBP were similarly downregulated, whereas UQCRH was upregulated, following the two exercise training regimens. As a major subunit of mitochondrial complex III, UQCRH is responsible for electron transfer between cytochrome *c* and cytochrome *c*1 during oxidative phosphorylation. As a catalytic subunit of pyruvate dehydrogenase phosphatase (PDP) identified in mammalian mitochondria, PDP1 is a serine phosphatase that catalyzes the dephosphorylation of pyruvate dehydrogenase, which catalyzes the oxidative decarboxylation of pyruvate to produce acetyl-CoA and NADH, linking glycolysis to the TCA cycle and lipogenic pathways. OSBP, a lipid binding protein, acts as a cholesterol-dependent scaffold for protein phosphatases in the negative regulation of the extracellular signal-regulated kinase signaling pathway [42], which is required for preventing dysfunctional mitochondria induced by oxidative stress in the skeletal muscle [43]. Figure 5 shows these pathways and their interactions.

### Autophagy

Recent studies have revealed that autophagy actually maintains muscle mass and that its function declines during muscle aging [44]. Conversely, autophagy inhibition leads to loss of muscle strength and dysfunction of OXPHOS and induces a maladaptive stress response responsible for myofiber atrophy in the aged [45]. Exercise training has been reported to boost selective autophagy, which may prevent age-induced declines in muscle mass and mitochondrial fragmentation [46]. We identified Sec22b, ADP, and Ctsd as altered proteins related to autophagy in aged skeletal muscles following the HIIT protocol. Ctsd, a lysosomal marker, promotes enhanced cargo clearance via an increase in lysosomal capacity. The enhanced expression of lysosomal Ctsd coincided with an elevated LC3-II/I protein ratio and expression of Beclin 1, ATG3, and LC3-II protein in aged skeletal muscle, all of which are indicative of maintained or even elevated formation of autophagic vacuoles and ongoing lysosomal degradation. Indeed, LC3-II, which participates in maturation of the phagophore and/or autophagosome, is converted from LC3-I by covalently bonding to phosphatidylethanolamine via ATG7- and ATG3-dependent conjugation events. Meanwhile, Sec22, a key regulator of the secretory pathway, facilitates ATG9 recruitment to the phagophore assembly site and is therefore required for autophagosome formation [47]. However, the current observation conflicts with a study reporting a decreased LC3-II/I ratio in the triceps [48] and quadriceps [49] muscles of aged mice with exercise training compared to that in untrained aged mice. However, they are consistent with previous findings that autophagic activity increases in gastrocnemius muscles with resistance training [46] and downhill/uphill training [6] in aged rats, and support the hypothesis proposed by Kim et al. that the expression of autophagy molecules is highly dependent on muscle type and exercise type [6].

A previous study has indicated that exercise training can improve aging-related impairments in muscle strength and autophagic activity via adiponectin/AdipoR1 axis-mediated AMPK/FOXO-dependent mechanisms in senescence-accelerated P10 mice [50]. In corroboration of these findings, the current observation of increased ADP, ADPR1, p-AMPK, Beclin 1, and p-FOXO1 expression is consistent with the activation of ADP/ADPR1 and FOXO signaling pathways based on KEGG analysis in aged skeletal muscles from the HIIT but not the MICT group of rats; it is thus reasonable to hypothesize that HIIT enhances ADP-induced AMPK signaling activation. Indeed, active AMPK facilitate the expression of several autophagy-related genes by directly phosphorylating Beclin 1 and FOXO1. Taken together, these results suggest that HIIT initiated late in life offered more benefits in stimulating the ADP/ADPR1 axis compared to MICT, which may improve aging-related impairments in autophagy via an AMPK/FOXO-dependent mechanism, and may therefore contribute to improving grip power in aged rats.

### Study limitations

The present study was designed to identify differences in aged skeletal muscle protein compositions between rats undergoing HIIT and MICT compared with the SED group. However, there are limitations to the chosen study design. First, although animal models are important to preclinical trials, substantial work is needed in preclinical and human studies to fully characterize the different benefits of HIIT and MICT in the elderly. Second, protein expression analysis does not enable identification of differences in the activities of proteins in the DEP datasets due to differences between the nuclear and cytoplasmic expression of proteins. Third, we reported data only from the red gastrocnemius muscle; there is obviously no comparison with another muscle fiber type such as extensor digitorum longus, tibialis anterior, and soleus muscle. Notably, several studies have shown that both the soleus and red gastrocnemius muscle were similar as characterized by mitochondrial decline and decrease in anti-apoptotic factors at old age and increased oxidative stress constitutes a common causative factor for sarcopenia [51]; however differential protein profile adaptations of skeletal muscles and fiber switch to exercise training may vary with regard to muscle fiber type and need to be determined. Fourth, our study was restricted to female rats; however, we cannot exclude that the protein profiles might differ in male rats. The prevalence and severity of sarcopenia differs between genders, starting earlier in females but progressing faster in males [52], which may be attributed at least partially by the differential influences of male and female sex hormones. Indeed, the age associated decline in estrogen is more prominent in females, where a 90% fall in serum estradiol occurs across menopause [53], compared to a fairly constant decline in testosterone of 1.6% per year in men that begins in the late third or early fourth decade [54]. Previous work has suggested that muscle mass and strength gains following prolonged exercise training are greater in older women compared to those in older men [55]. When considering translational significance and practical implications, we used female rats at 18 months of age (pre-sarcopenic) in the present study, as they have been shown to better reflect the changes in estrogen metabolism during exercise training (though this data was not shown), which may contribute to accelerate age-related muscle and bone wasting. Lastly, although both protocols implicated common and specific pathways in aged rats, the exact mechanisms could not be determined directly, especially as there was a lack of evidence from animals with specific gene knockouts to clarify whether the effects of the two exercise models on the FOXO and adipocytokine signaling pathways were direct or in association with a signaling cascade.

## METHODS

### Animals

Thirty-six female Sprague-Dawley rats (8 months old) were purchased from Guangdong Medical Laboratory Animal Center (Guangdong, China) and raised under an artificial 12-h light-dark cycle (6:00 AM–6:00 PM) at constant room temperature (23 ± 1°C) in the Laboratory Animal Center, School of Sports Science and Physical Education, South China Normal University. Animals were housed in their respective groups in a collective cage and received water and standard laboratory chow (56.8% carbohydrate, 22.5% protein, 3.5% lipids, and 17.2% other nutrients) *ad libitum*. Every possible effort was made to minimize both the number and suffering of used animals, according to the principles of the Declaration of Helsinki. The protocols were approved by our institution's regional Ethics Committee for animal studies.

At 18 months of age, all rats were divided into three groups: sedentary (SED, *n =* 12), MICT (*n* = 12), and HIIT (*n* = 12). SED group rats were handled in an identical manner to the treadmill running groups but did not participate in any exercise treatment for the 8 months of the experiment. During the experiment, 12 rats were excluded from the study: two SED rats (owing to severe eye infections), four MICT rats (two rats died from exercise-unrelated causes and two rats were excluded owing to severe claw infections), and six HIIT rats (two rats owing to nonadherence to the training protocol and four rats because of severe claw and tail infections).

### Training protocol

All rats were trained over the course of a 2-week acclimatization to run on an adapted motor-driven treadmill designed for rats (model FD000043; Flyde Apparatus, Guangzhou, China). During the first week of training, rats were individually placed into a treadmill lane at a 0° incline for a total of 10 min. At the start of the second week, rats ran at 10 m·min^–1^, progressing to 15 cm/s at a 0° incline for 30 min by the end of the week. Maximal oxygen uptake (VO_2max_) was determined as reported previously [7].

MICT included 1 min of warm-up at a constant running speed of 10 m·min^–1^, which corresponded to 35–40% of VO_2max_, followed by 45 min of constant running at 17 m·min^–1^ (75–80% VO_2max_) and cool-down at a constant speed of 10 m·min^–1^ for 1 min. HIIT included 4 min of low-speed running at 15 m·min^–1^ (45–55% VO_2max_), followed by 1 min of high-speed running at 25 m·min^–1^ at a 0% grade (9 repetitions; 90–95% VO_2max_) and cool-down at a constant speed of 10 m·min^–1^ for 1 min. Therefore, the total volume for the HIIT group was 3925 m·week^–1^, and the average intensity was about 75–80% VO_2max_, matching that for the MICT group. To prevent avoidance and ensure exercise training, rats received a light electrical shock (6 mA) if they sat at the base of the treadmill. Within 2 weeks of training, the rates of exercise avoidance were minimal, and electrical shock was no longer needed. Exercise training sessions were held at the same time each morning.

After 24 h of rest following the final training, all rats underwent orderly inclined plane, grip power, and exercise tolerance tests within one week. After exercise tolerance test, rats were fasted for 48 h and anesthetized with 50 mg·kg^−1^ body weight pentobarbital sodium by intraperitoneal injection. The red portion of the gastrocnemius muscle was rapidly excised and immediately frozen in liquid nitrogen. Samples were subsequently stored at -80°C until analysis.

### Physical performance measures

All assessments were performed by the same investigator, who was blinded to the identity of the mice. Additionally, different assessments were performed in the same order over the course of one week at the baseline and endpoint, and each was performed at the same time of the day. All rats underwent physical performance assessment, including endurance, progressive running, inclined plane, and grip power tests. Further details on these tests are reported previously [5, 6].

### Histochemical analysis

The gastrocnemius muscle was cryosectioned and labeled with hematoxylin and eosin (HE). Transverse sections of 12 μm were prepared using a cryostat at -20°C. Slides were stained with HE, mounted in Permafluor (Invitrogen, Carlsbad, CA), and imaged using a microscope (Olympus BX51 microscope, Olympus, Tokyo, Japan). The total cross-sectional area (CSA) and frequency distribution of fiber CSA were measured on 400 fibers in each animal, and the mean was calculated. HE staining of gastrocnemius muscles was performed as reported previously [6].

### Proteomics

The detailed sample preparation and proteolytic digestion protocols are reported previously [4]. Samples were subjected to LC-MS/MS. Label-free LC-MS/MS data were acquired using a high-resolution QExactive MS (Thermo Fisher Scientific) [4]. The acquired raw data were analyzed using MaxQuant software and the implemented Andromeda software (1.5.2.8 and 1.5.3.8). The false discovery rate (FDR) was set to <1% at both the peptide spectrum match (PSM) and protein levels during searching and was automatically calculated by the proteome Discoverer software [9, 10]. As the protein quantification algorithm relies on the extracted ion chromatograms of unique peptides, we selected proteins with ≥ 2 unique peptides for further label-free analysis. The inclusion criteria for protein identification were based on the presence of the protein in at least two of three replicates and in four of six animals analyzed per group. Differentially expressed proteins (DEPs) between the two experimental groups were defined as those with a *p* < 0.05 and a fold change > 1.5 compared to that in the SED group [9].

To categorize the cellular components (CC), biological processes (BP), and molecular functions (MF) of the identified statistically significant DEPs in our data set, we imported the DEPs into Gene ontology (GO), and the Kyoto Encyclopedia of Genes and Genomes (KEGG) pathway analysis. The enriched KEGG pathway mapping was perform on KEGG (2.0, http://www.genome.jp/kegg/), a database resource with large-scale datasets obtained from high-throughput experimental technologies designed to understand high-level functions and utilities of biological systems [4, 9].

### Immunoblotting analysis

Protein expression was assayed using western blotting as described previously [4]. Gastrocnemius muscle tissue was homogenized in tissue-lysing buffer. The supernatant from centrifugation was used for subsequent western blotting on a standard 10% SDS-PAGE gel on a Bio-Rad electrophoresis system (Hercules, CA, USA). Horseradish peroxidase-conjugated donkey anti-rabbit IgG (H+L; 711-035-152, Jackson ImmunoResearch Europe, Newmarket, UK) was used as the secondary antibody, incubated for 1 h at room temperature, and washed with TBS-T. Bands were visualized using the Enhanced Chemiluminescence Detection Kit (Amersham Pharmacia Biotech, Little Chalfont, UK). Chemiluminescence signal was captured using the ChemiDoc MP Imaging System (Bio-Rad), and digital images were generated. Resulting images were quantified using ImageJ software (NIH, Bethesda, MD, USA). All antibodies used for western blotting are listed in Supplementary Table 1.

### Quantitative PCR

mRNA expression was assayed using reverse transcription-polymerase chain reaction (RT-PCR) as described previously [3]. Total RNA was isolated from red gastrocnemius muscle tissues using TRIzol® (Life Technologies). Random primers and GoScript reverse transcriptase (Promega) were used to generate cDNA. RT-PCR analyses were performed on a CFX Connect Real-Time system using iTaq Universal SYBR Green Supermix (Bio-Rad). The data were normalized to *Gapdh* expression to generate a ΔCt value, which was then normalized to the mean ΔCt of the SED group. All primers used for quantitative PCR analyses are listed in Supplementary Table 2.

### Statistical analysis

Statistical analysis was performed using GraphPad Prism 6.0 software (GraphPad Software, San Diego, CA, USA). Values are presented as the mean ± standard deviation. Prior to analysis, all data were checked for normality using the one-sample Kolmogorov-Smirnov test. Differences among SED, MICT, and HIIT groups were assessed via one-way analysis of variance followed by Tukey’s post-hoc test. Comparisons between two groups were carried out using Student’s *t*-test. Differences with p-values < 0.05 were considered significant.

## CONCLUSIONS

In summary, our study showed that both HIIT and MICT differentially altered the protein abundance in skeletal muscle in aged rats. Changes in proteins related to skeletal muscle contraction, cell integrity, mitochondrial metabolism, apoptosis and regeneration, and anti-oxidation demonstrated the importance of the HIIT and MICT protocol as a strategy for reducing the deleterious effects inherent to biological aging. These results provide important information that improves our understanding of the mechanisms underlying FOXO1 pathways and explores their association with pathological alterations after long-term exercise training. Moreover, HIIT prevented the aging-related impairment of autophagy, possibly via an AMPK-dependent mechanism mediated by the adiponectin/AdipoR1 axis, thus improving the age-related loss of muscle mass and grip power in aged rats.

## Supplementary Material

Supplementary Tables
