# Correction for: Proteomics-based identification of different training adaptations of aged skeletal muscle following long-term high-intensity interval and moderate-intensity continuous training in aged rats

**DOI:** 10.18632/aging.102596

**Published:** 2019-11-30

**Authors:** Fang-Hui Li, Lei Sun, Da-Shuai Wu, Hao-En Gao, Zhu Min

**Affiliations:** 1 School of Sport Sciences, Nanjing Normal University, Nanjing, China

**Keywords:** correction

Original article: Aging. 2019; 11:4159–4182. . https://doi.org/10.18632/aging.102044

**This article has been corrected:** The authors requested to replace Figure 4C that was incorrectly prepared due to mistake in the organization of this paper. More specifically, the PGC-1 protein band in the western blot being was placed on the COX-IV band. The authors declare that this is was result of their negligence and was not intentional. This correction does not change the results or conclusions of this paper. The corrected version of Figure 4 is provided below.

**Figure 4:**
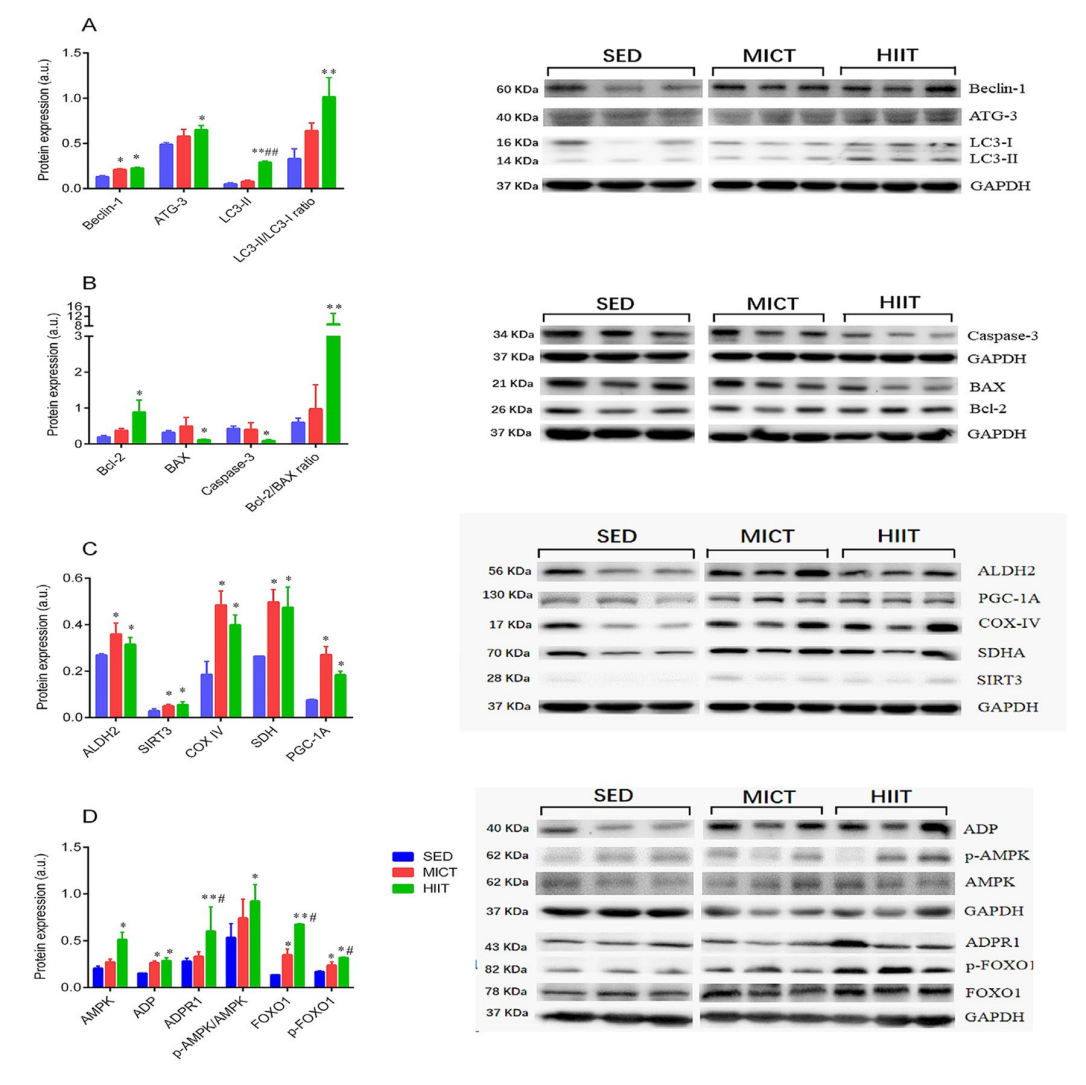
Expression of autophagy (**A**), apoptosis (**B**), and mitochondrial function markers (**C**), and adipocytokine signaling-related proteins (**D**). SED, sedentary; MICT, moderate-intensity continuous training; HIIT, high-intensity interval training; succinate dehydrogenase, SDH; sirtuin 3, SIRT3; aldehyde dehydrogenase 2, ALDH2; peroxisome proliferator-activated receptor γ coactivator-1Α, PGC-1a; adiponectin, ADP; autophagy-related gene-3, Atg-3; microtubule-associated protein 1 light chain 3 II, LC3-II; B-cell lymphoma 2, Bcl-2; Bcl-2-associated X protein, Bax; AMP-activated protein kinase, AMPK; adiponectin receptor 1, ADPR1; Forkhead box O1, FOXO1. Data were analyzed by one-way ANOVA followed by Tukey’s post-hoc and are presented as mean ± SD. * p < 0.05 *vs*. SED; ** p < 0.01 *vs*. SED; ^#^ p < 0.05 *vs*. MICT; ^##^ p < 0.01 *vs*. MICT.

