# Supplementary material for: Proteomics-based identification of different training adaptations of aged skeletal muscle following long-term high-intensity interval and moderate-intensity continuous training in aged rats
Source: Aging (Albany NY). 2019 Jun 26;11(12):4159–82. doi: 10.18632/aging.102044 (PMC11623340; doi:10.18632/aging.102044)
Supplement: Supplementary Tables [file aging-11-102044-s001.pdf]

SUPPLEMENTARY MATERIALS

Supplementary Tables

Supplementary Table 1. All antibody used for western blot analysis.

Target	Manufacturer	Product	Primary
ALDH2	Abcam	ab108306	1:1000
Sirtuin 3	CST	2627	1:1000
COX-IV	CST	4844	1:1000
SDH	CST	11998	1:1000
LC3A/B	CST	12741	1:1000
ATG-3	CST	3415	1:1000
Beclin-1	CST	3495	1:1000
Adiponectin Receptor 1	Abcam	ab126611	1:1000
Adiponectin	Abcam	ab22554	1:1000
Bax	Abcam	ab32503	1:500
Bcl-2	Abcam	ab196495	1:500
Caspase-3	Abcam	ab13847	1:500
FOXO1	Abcam	ab70382	1:1000
p-FOXO1	Abcam	ab131339	1:1000
GAPDH	CST	2118	1:1000

Supplementary Table 2. The primer sequences used in reverse transcription-polymerase chain reaction.

Primers	Forward (5′-3′)	Reverse (5′-3′)
Dnaja2	AAGAGGCGAGGACATGATGC	CATAATGCGCACACCTCGAC
Calnexin	GAATAGAATGCGGTGGTGC	TGTGGCGAA AGATGA AATG
Sypl2	TACCTGCGCTTCCACAAAGT	GCAGCAACCAGCCAAAAGAA
JP1	TCCTGAGTCAAGTCCCAAAC	CCTCTGTCATCCTCCCTGT
SOD2	AGGGCCTGTCCCATGATGTC	AGAAACCCGTTTGCCTCTACTGAA
FOXO1	TCCTCGAACCAGCTCAAACG	GGCGGTGCAAATGAATAGCAAG
ADP	GAGACGCAGGTGTTCTTGGT	GGAACATTGGGGACAGTGAC
ADPR1	GTTGTACCCACCATGCACTTT	GGTTGGACACCCCATAGAAGT
GAPDH	TGCCGCCTGGAGAAACCTGC	TGAGAGCAATGCCAGCCCCA

Supplementary Table 3. Proteins with significantly altered abundance in red gastrocnemius between MICT and SED groups.

Protein IDs	Dscription	Function	Uniquepetides	Folds	*p*-value
**Muscle contraction**					
A0A0G2K1V4	myosin heavy chain 1, GN = Myh1	muscle contraction	25	2.75	0.047
D4A6M0	synaptophysin-like 2, GN = Sypl2	calcium ion homeostasis	3	2.20	0.027
D3ZQ55	junctophilin 1, GN = Jph1	calcium-release channel activity	10	1.66	0.035
A0A0G2K3E0	aquaporin 1, GN = Aqp1	maintenance of actin cytoskeleton polarity	2	1.81	0.046
Q6IMZ5	tropomodulin 1, GN = Tmod1	muscle contraction	11	0.62	0.046
D3ZGP9	dehydrogenase 7C, GN= Dhrs7c	regulation of release of calcium ion	12	0.46	0.046
**Focal adhesion signaling**					
D4A3B0	talin 2, GN = Tln2	Actin filament binding	8	3.82	0.046
D3ZRN3	actin beta-like 2, GN = Actbl2	ATP binding	3	2.49	0.046
A0A0G2K013	actinin alpha 4, GN = Actn4	actin filament	2	3.28	0.029
A0A0G2K3C8	nidogen 2, GN = Nid2	plasma membrane	5	0.56	0.047
Q6AY56	tubulin alpha 8, GN= Tuba8	microtubule cytoskeleton organization	5	0.41	0.027
**Mitochondrial metabolism**					
Q6PCT8	succinate dehydrogenase complex subunit D,GN = Sdhd	TCA, succinate dehydrogenase complex	2	2.14	0.046
Q5M9I5	ubiquinol-cytochrome c reductase hinge protein,GN = Uqcrh	ubiquinol-cytochrome-c reductase activity	3	1.95	0.035
D4A565	NADH:ubiquinone oxidoreductase subunit B5,GN = Ndufb5	mitochondrial respiratory chain complex I	5	1.77	0.046
Q66HP8	solute carrier family 25 member 20,GN = Slc25a20	mitochondrial carnitine transport	10	1.60	0.035
D3ZD09	cytochrome c oxidase subunit 6B1, GN = Cox6b1	mitochondrial TCA, cytochrome c oxidase	6	1.53	0.046
A1L1J4	pyruvate dehyrogenase phosphatase catalytic subunit 1, GN = Pdp1	mitochondrion serine/threonine phosphatase activity	6	0.61	0.046
**Protein processing in endoplasmic reticulum**					
D3ZE72	methionyl aminopeptidase 1, GN = Metap1	proteolysis	3	2.12	0.027

Q5M9H7	DnaJ heat shock protein family (Hsp40) member A2, GN = Dnaja2	unfolded protein binding, chaperone	5	1.91	0.046
P35565	Calnexin, GN = Canx	unfolded protein binding	5	0.61	0.050
Q5XIP0	DnaJ heat shock protein family (Hsp40) member B4, GN = Dnajb4	unfolded protein binding, chaperone binding	7	0.51	0.035
**Apoptotic and regeneration**					
G3V7U0	cysteine and glycine rich protein 3, GN = Csrp3	positive regulation myoblast differentiation	7	2.78	0.046
D3ZUC1	smoothelin-like 2, GN = Smtnl2	myotubes differentiating	5	1.55	0.046
F1M8K	dystroglycan 1, GN = Dag1	skeletal muscle tissue regeneration	9	1.53	0.046
A0A0H2UHF6	ADP-ribosylation like GTPase 6 interacting protein 5, GN = Arl6ip5	negative regulation of apoptotic	4	1.51	0.046
Q6AXS5	Serpine1 mRNA binding protein 1, GN = Serbp1	regulation of apoptotic process	3	0.44	0.047
**Anti-oxidation**					
P07895	superoxide dismutase 2, GN = Sod2	age-dependent response to ROS	8	1.55	0.046
P51635	aldo-keto reductase family 1 member A1, GN = Akr1a1	aldehyde catabolic process	4	0.60	0.046
R4GNK3	thioredoxin 1, GN = Txn1	thioredoxin-disulfide reductase activity	3	0.35	0.047

Note. Student's t-test, corrected for false discovery rate with Benjamini-Hochberg method was applied to normalized expression data to identify DEPs. $p < 0.05$ was set as the nominal significance cutoff, resulting in 29 proteins. The ratio MICT/SED represents the fold change in protein expression levels between the MICT and SED groups, reflecting proteins that were upregulated (> 1.5) or downregulated (< 0.677) in the MICT group.

Supplementary Table 4. Proteins with significantly altered abundance in red gastrocnemius between HIIT and SED groups.

Protein IDs	Description	Function	Unique petides	Folds	*p*-value
**Muscle contraction**					
D4A6M0	synaptophysin-like 2, GN = Sypl2	cellular calcium ion homeostasis	3	1.95	0.023
P0DP29	calmodulin 2, GN = Calm2	negative regulation of calcium-release channel activity	11	1.65	0.031
D3ZQ55	junctophilin 1, GN = Jph1	calcium-release channel activity	10	1.61	0.027

A0A140TAF0	tropomyosin 3, GN = Tpm3	muscle contraction	7	0.58	0.034
**Focal adhesion signaling**					
D3ZRN3	actin, beta-like 2, GN = Actbl2	ATP binding	3	2.64	0.027
P60711	actin, beta, GN = Actb	ATP binding	8	1.50	0.018
**Mitochondrial metabolism**					
F1LZW6	solute carrier family 25 member 13, GN = Slc25a13	L-glutamate transmembrane transporter activity	2	2.29	0.032
Q5M9I5	ubiquinol-cytochrome c reductase hinge protein, GN = Uqcrh	ubiquinol-cytochrome-c reductase activity	3	1.79	0.024
P11662	mitochondrially encoded NADH dehydrogenase 2, GN = Mt-nd2	NADH dehydrogenase (ubiquinone) activity	2	1.70	0.027
O88989	malate dehydrogenase 1, GN = Mdh1	malate dehydrogenase activity, TCA	14	1.52	0.027
P25409	glutamic--pyruvic transaminase, GN = Gpt	L-alanine:2-oxoglutarate aminotransferase activity	15	1.51	0.024
P13221	glutamic-oxaloacetic transaminase 1, GN = Got1	negative regulation of collagen biosynthetic process	23	1.52	0.027
A1L1J4	pyruvate dehyrogenase phosphatase catalytic subunit 1, GN = Pdp1	mitochondrion serine/threonine phosphatase activity	6	0.64	0.034
D4A9D8	oxysterol binding protein, GN = Osbp	sterol transporter activity	3	0.55	0.027
**Apoptotic and regeneration**					
Q5XIG8	serine/threonine kinase receptor associated protein, GN = Strap	negative regulation of TGF beta receptor pathway	6	1.57	0.040
D3ZUC1	smoothelin-like 2, GN = Smtnl2	myotubes differentiating	5	1.51	0.034
F1M454	tubby-like protein 3, GN = Tulp3	phosphatidylinositol binding	2	1.77	0.034
A0A0G2K654	histone cluster 1 H1 family member c, GN = Hist1h1c	cooperates with various proapoptotic proteins	2	0.63	0.034
G3V9M6	fibrillin 1, GN = Fbn1	skeletal muscle system development	33	0.67	0.042
**Autophagy**					

A0A0G2K845	adiponectin, GN = ADP	adiponectin-activated signaling pathway	2	2.50	0.027
Q6P6T6	SEC22 homolog B, vesicle trafficking protein, GN = Sec22b	autophagosome assembly	8	1.56	0.024
**Anti-oxidation**		negative regulation of autophagosome assembly	3	0.55	0.024
P07895	superoxide dismutase 2, GN = sod2				
P08009	glutathione S-transferase mu 2, GN = Gstm2	age-dependent response to ROS	8	2.00	0.027
**Other**			3	1.54	0.024
F1M013	ribosomal protein L7a, GN = Rpl7a				
Q4V8F6	poly(rC) binding protein 2, GN = Pcbp2	ribosome biogenesis	7	0.55	0.034
		C-rich single-stranded DNA binding	2	0.45	0.027

Note. Student's t-test, corrected for false discovery rate with Benjamini-Hochberg method was applied to normalized expression data to identify DEPs. $p < 0.05$ was set as the nominal significance cutoff, resulting in 26 proteins. The ratio HIIT/SED represents the fold change in protein expression levels between the HIIT and SED groups, reflecting proteins that were upregulated (> 1.5) or downregulated (< 0.677) in the HIIT group.

Supplementary Table 5. Proteins involved in KEGG pathways significantly enriched in the MICT/HIIT groups.

Map_ID	Map_name	Protein name	Trend	*p* value	FDR	Enrichment Factor
**MICT *vs.* SED**						
**rno04141**	Protein processing in endoplasmic reticulum	DnaJ heat shock protein family (Hsp40) member A2	Down	0.037	0.590	0.180
		Calnexin				
		DnaJ heat shock protein family (Hsp40) member B12				
		Sec23 homolog A, coat complex II component				
		Ribophorin II				
**rno04068**	FOXO signaling pathway	Superoxide dismutase 2	Over	0.014	0.500	0.310
		Solute carrier family 2 member 4				
		Growth factor receptor bound protein 2				
**HIIT *vs* SED**		Mitogen activated protein kinase kinase 1				
**rno04920**	Adipocytokine signaling pathway	Adiponectin	Over	0.036	0.580	0.270
		Solute carrier family 2 member 4				
		Acetyl-CoA carboxylase beta				
**rno04068**	FOXO signaling pathway	Superoxide dismutase 2	Over	0.050	0.580	0.230
		Solute carrier family 2 member 4				
		Homer scaffold protein 1				
